# Supplementary figures and images for: Prognostic Values of Tissue and Serum Angiogenic Growth Factors Depend on the Phenotypic Subtypes of Colorectal Cancer
Source: Cancers (Basel). 2023 Jul 29;15(15):3871. doi: 10.3390/cancers15153871 (PMC10417397; doi:10.3390/cancers15153871)

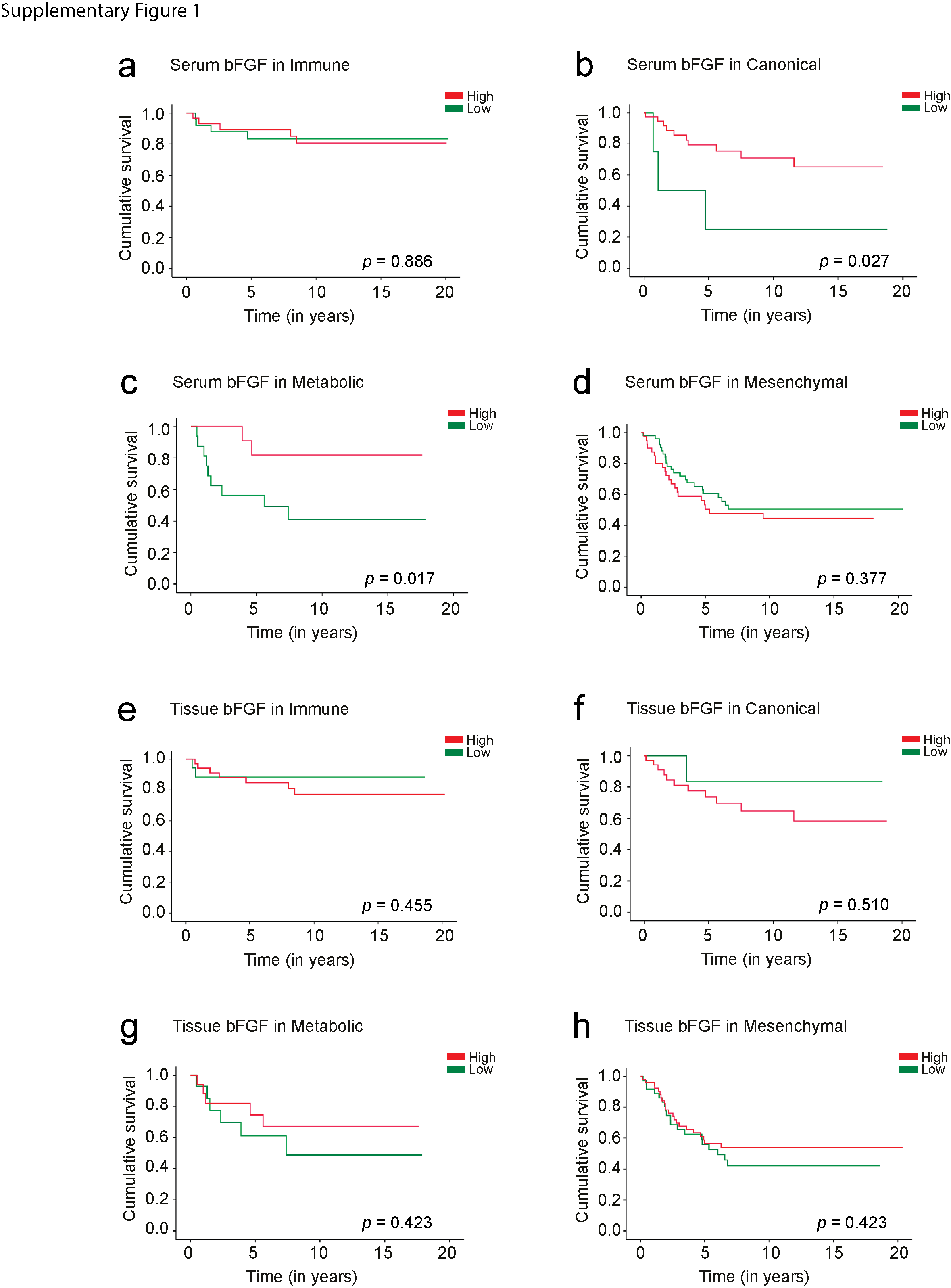

Supplement: Supplementary file 1 [file cancers-15-03871-s001.zip › FigS1.png]

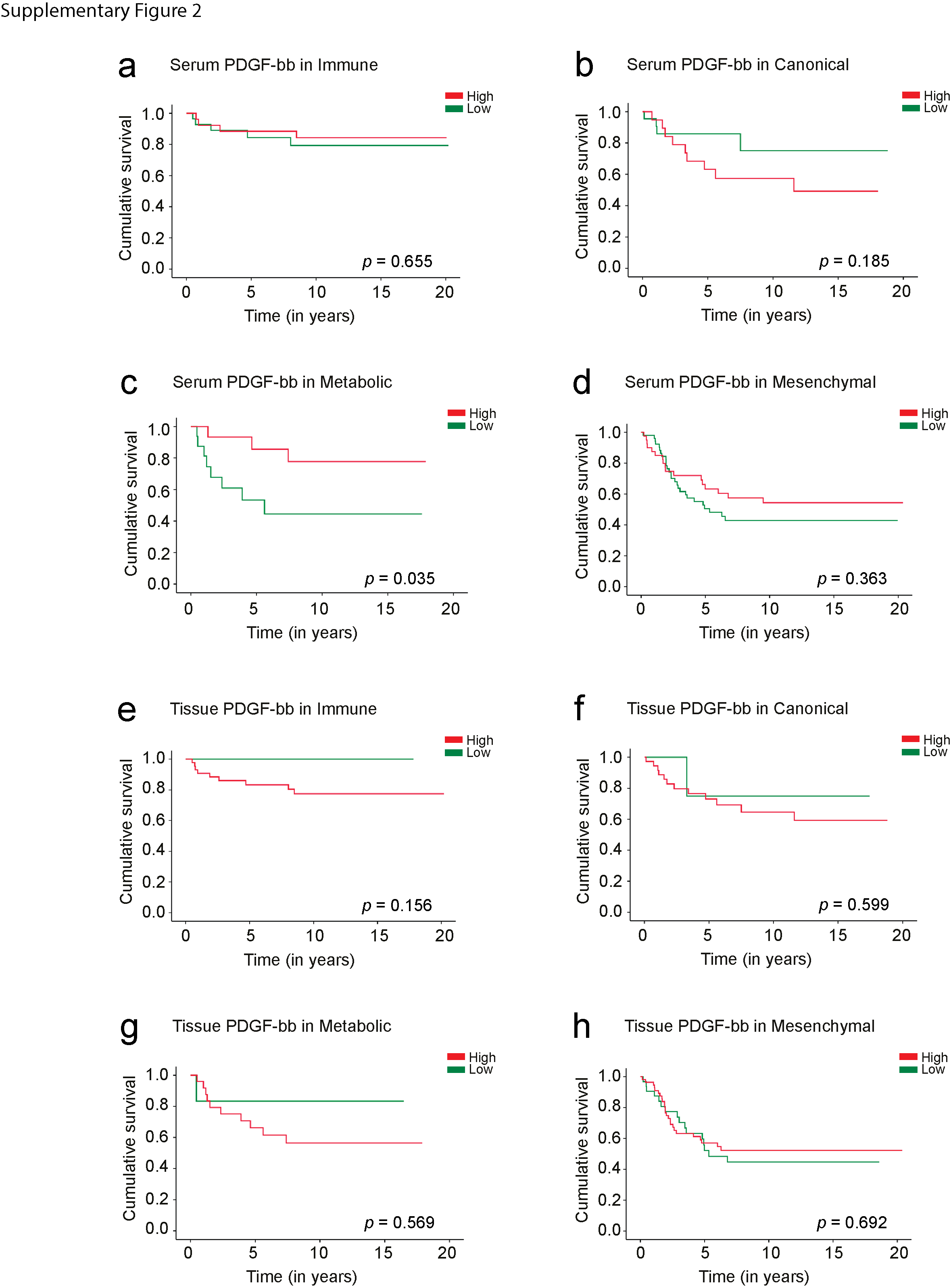

Supplement: Supplementary file 1 [file cancers-15-03871-s001.zip › FigS2.png]
